# Elevated systemic immune-inflammatory index predicts poor coronary collateralization in type 2 diabetic patients with chronic total occlusion

**DOI:** 10.3389/fcvm.2024.1490498

**Published:** 2024-12-12

**Authors:** Lin Shuang Mao, Yi Xuan Wang, Zhi Ming Wu, Feng Hua Ding, Lin Lu, Wei Feng Shen, Yang Dai, Ying Shen

**Affiliations:** ^1^ Department of Cardiovascular Medicine, Rui Jin Hospital, Shanghai Jiao Tong University School of Medicine, Shanghai, China; ^2^ Institute of Cardiovascular Diseases, Shanghai Jiao Tong University School of Medicine, Shanghai, China

**Keywords:** systemic immune-inflammation index, coronary collateral circulation, type 2 diabetes mellitus, stable coronary artery disease, chronic total occlusion

## Abstract

**Objective:**

This study compared the value of different systemic immune-inflammatory markers for evaluating coronary collateralization (CC) in patients with type 2 diabetes mellitus (T2DM) and chronic total occlusion (CTO).

**Methods:**

Systemic immune-inflammation index (SII), systemic inﬂammation response index (SIRI) and pan-immune-inﬂammation value (PIV) were calculated at admission in 1409 T2DM patients with CTO. The degree of coronary collaterals was estimated using the Rentrop scoring system and categorized into poor (Rentrop score 0 or 1) or good (Rentrop score 2 or 3) CC. The predictors of poor CC were determined by multivariate regression analysis, and the diagnostic potential of these indexes was analyzed by Receiver Operating Characteristic (ROC) curves.

**Results:**

SII, SIRI and PIV levels increased stepwise across Rentrop score 0–3, with significantly higher levels in patients with poor CC than in those with good CC (*P* < 0.001). After adjusting for confounders, SII, SIRI and PIV (per tertile) remained independent factors for poor CC. SII predicted poor CC better than SIRI and PIV (AUC: 0.758 vs. 0.680 and 0.698, all *P* < 0.001). There existed an interaction between blood concentration of HbA1c and SII (*P* < 0.001), with high SII levels being associated with a greater risk (OR: 5.058 vs. 2.444) and providing a better predictive ability for poor CC (AUC: 0.817 vs. 0.731) in patients with HbA1c < 6.5% compared to those with HbA1c ≥ 6.5%.

**Conclusion:**

Our study shows that elevated SII provides a better prediction for poor CC in T2DM patients with CTO especially at good glycemic control.

## Introduction

Coronary collateralization (CC) is an adaptive response to transient or permanent coronary artery occlusion (1, 2). The clinical relevance of the status of CC has been extensively investigated, showing that robust coronary collaterals are frequently associated with a favorable outcome by protecting ischemic myocardium, improving cardiac function, and decreasing future cardiovascular events and mortality (3, 4). Collateral formation is a complex multi-step process involving an array of pro-angiogenic and anti-angiogenic factors (5). Type 2 diabetes mellitus (T2DM) is considered as a major risk factor for severe and diffuse coronary atherosclerosis and poor clinical outcome, and T2DM patients with chronic total occlusion (CTO) are more prone to develop poor CC compared to non-diabetic counterparts (6, 7). Previous studies demonstrated that chronic low-grade inflammation, along with immune dysregulation, is a common mechanism of both atherosclerosis and T2DM, which may lead to impaired arteriogenesis and angiogenesis as well as new vessel growth in response to ischemia (8).

Systemic immune-inflammation index (SII), systemic inflammation response index (SIRI) and pan-immune-inﬂammation-value (PIV) have been emerged as novel markers by integrating three subtypes of white blood cell and platelets and indicate the balance between the inflammatory response and immune status (9). Numerous studies have suggested that SII and SIRI are associated with atherogenesis and cardiovascular outcome (9–11). Recently, Kelesoglu (10), Adali (12) and co-workers reported that high SII levels were correlated significantly with poor CC in patients with CTO. Yilmaz et.al demonstrated that PIV independently predicted poor CC in stable coronary artery disease patients (13). However, the value of systemic immune-inflammatory markers in the evaluation of angiographic CC in patients with T2DM and CTO remains unclear, mostly because of the small sample size and heterogenous population in the previous studies. Here, we investigated the relationship of SII, SIRI and PIV to coronary collateral formation in a large cohort of T2DM patients with CTO. The predictive value of elevated SII, SIRI and PIV for poor CC was also compared in relation to the status of glycemic control.

## Methods

This study was part of the COLLECT study (**CO**ronary Co**LL**ateralization in Type 2 diab**E**tic Patients with **C**hronic **T**otal Occlusion) registered with ClinicalTrials.gov (NCT06054126), which aimed to explore the risk factors and treatment options for poor collateralization in T2DM patients with CTO. All participants provided written informed consent. The study protocol was approved by the Ethics Committee of Shanghai Ruijin Hospital, Shanghai Jiao and the study was conducted in accordance with the Declaration of Helsinki.

### Study population

**Figure 1:**
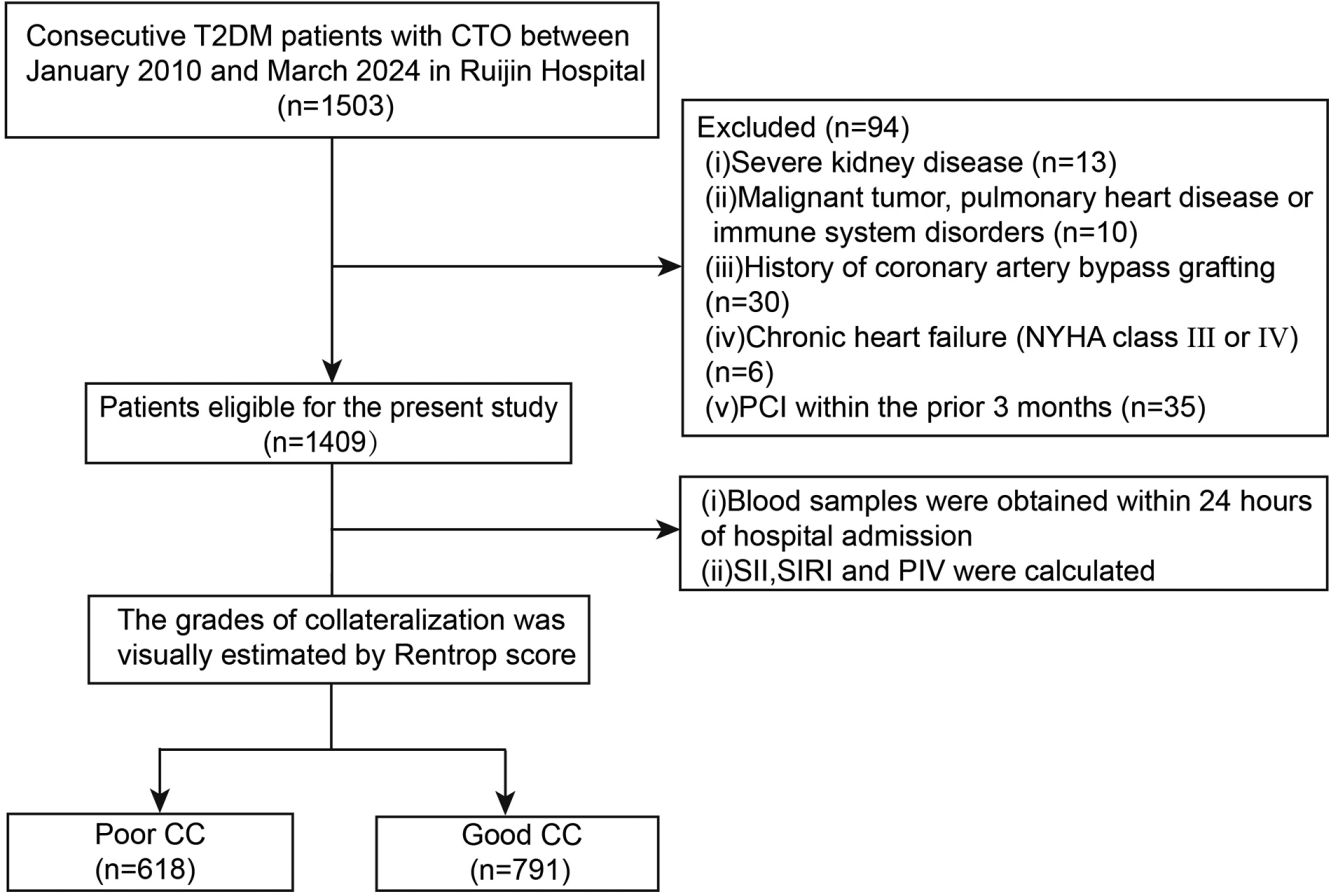
Flowchart of the study. T2DM type 2, diabetes mellitus; CTO, chronic total occlusion; PCI, percutaneous coronary intervention; SII, systemic immune-inflammation index; SIRI, systemic inflammation response index; PIV, pan-immune-inﬂammation value; CC, coronary collateralization.

A total of 1,503 consecutive stable angina patients who had T2DM between January 2010 and March 2024 were screened. All patients were ≥18 years in age and had at least one lesion with angiographic 100% occlusion in major epicardial coronary arteries for more than 3 months. This angiographic inclusion was used based on well-established knowledge that a severe coronary artery obstruction was a prerequisite for spontaneous collateral recruitment. For the purpose of the study, 94 patients were excluded because of severe chronic kidney disease requiring dialysis (*n* = 13), malignant tumor and pulmonary heart disease or immune system disorders (*n* = 10), history of coronary artery bypass grafting (*n* = 30), severe chronic heart failure (New York Heart Association functional class III or IV) (*n* = 6), or percutaneous coronary intervention (PCI) within the prior 3 months (*n* = 35). The remaining 1,409 T2DM patients with CTO were included in the final analysis (Figure 1).

Baseline clinical characteristics were collected from the Inpatient Medical Record Management Systems. Blood samples were obtained within 24 h of hospital admission, and hematological and biochemical data were measured by standard laboratory techniques. Laboratory personnel unaware of the patient's diagnoses analyzed the blood samples. SII, SIRI and PIV were calculated based on the following formula: SII = (neutrophil count × platelet count)/lymphocyte count; SIRI = (neutrophil count × monocyte count)/lymphocyte count; PIV = (monocyte count × neutrophil count × platelet count)/lymphocyte count (13, 14).

The diagnosis for T2DM was made according to the American Diabetes Association guidelines (14). Hypertension was diagnosed as the presence of office systolic blood pressure values ≥140 mmHg and/or diastolic blood pressure values ≥90 mmHg, or taking antihypertensive drugs for blood pressure control (15). Hypercholesterolemia was defined according to the Third Report of The National Cholesterol Education Program (NCEP) (16). Severe chronic kidney disease was defined as estimated glomerular filtration rate (eGFR) < 15 ml/(min·1.73 m^2^) (17).

### Coronary angiography and collateral grading

Coronary angiography was performed via the femoral or radial approach, and the degree of coronary artery narrowing was determined by quantitative coronary analysis (QCA) (18). Number of significant diseased coronary arteries (≥50% stenosis in major epicardial coronary artery) was used to assess coronary disease severity, and left main coronary stenosis was considered as 2-vessel disease (19).

Presence/absence and extent of collateral circulation were graded according to the Rentrop scoring system (20). Rentrop score 0 (no filling of any collateral vessels) and score 1 (filling of side branches of the artery by collateral vessels without visualization of the epicardial segment) were categorized into poor CC, whereas Rentrop score 2 (partial filling of the epicardial segment by collateral vessels) and score 3 (complete filling of the epicardial artery by collateral vessels) were classified into good CC. For patients with more than one chronic total occlusion, the vessel with the highest collateral grade was selected for analysis. Coronary collaterals were graded by two experienced interventional cardiologists blinded to patient's clinical characteristics. Any disagreement was resolved by a third reviewer.

### Statistical analysis

Continuous variables are presented as mean ± standard deviations (SD) or median (interquartile range, IQR), and were compared between groups by Student's *t*-test and Mann–Whitney *U* test for normally or non-normally distributed variables, respectively. Categorical variables are expressed as absolute number with percentage and were compared between groups by Chi-square test. One-way ANOVA analysis was performed to compare the difference of SII, SIRI and PIV between groups with 0–3 Rentrop score. To determine the independent predictors for poor CC, age, female gender, body mass index (BMI), hypertension, hypercholesterolemia, hematological data, eGFR and HbA1c together with SII, SIRI or PIV were adopted in multivariate logistic regression model 1–3, respectively. Receiver Operating Characteristic (ROC) analysis was made, and the predictive ability of SII, SIRI and PIV for poor CC in T2DM patients with CTO was evaluated by the area under the ROC curve (AUC). The Youden index was applied to find the optimal cutoff point for maximized sensitivity and specificity. The comparisons of AUCs were performed using DeLong test. All statistical analyses were done using SPSS version 26.0 (SPSS Inc., Chicago, IL, USA) and MedCalc software (Version 20.0.22; Medcalc Software, Mariakerke, Belgium). Statistical significance was set a two-tailed *P* value < 0.05.

## Results

### Baseline characteristics

Poor and good CC were detected in 618 (43.9%) and 791 (56.1%) patients, respectively. Compared to patients with good CC, those with poor CC were older and more female in gender, and had higher incidence of hypertension, hypercholesterolemia and prior myocardial infarction. As for laboratory measurements, platelet count, white blood cell count and neutrophil were higher, and fasting blood glucose (FBG), glycosylated hemoglobin (HbA1c), total cholesterol, low-density lipoprotein cholesterol (LDL-C) and blood urea nitrogen (BUN) were elevated, but monocyte, lymphocyte and eGFR were lower in patients with poor CC (*P* < 0.05 for all comparisons). The two groups did not significantly differ with respective to severity of coronary artery disease and medications (Table 1).

**Table 1:** Baseline characteristics of the study patients.

Variables	Poor CC (*n* = 618)	Good CC (*n* = 791)	*P* value
Age, years	66.1 ± 10.5	63.0 ± 11.0	<0.001
Female, No. (%)	159 (25.7)	139 (17.6)	<0.001
Body mass index, kg/m^2^	25.27 ± 3.01	25.03 ± 3.00	0.145
SBP (mmHg)	136.9 ± 21.9	135.8 ± 19.7	0.307
DBP (mmHg)	76.7 ± 12.3	77.2 ± 12.1	0.448
Smoking, No. (%)	240 (38.8)	298 (37.7)	0.659
Hypertension, No. (%)	472 (76.4)	555 (70.2)	0.009
Hypercholesterolemia, No. (%)	174 (28.2)	150 (19.0)	<0.001
Prior MI, No. (%)	128 (20.7)	130 (16.4)	0.044
Platelet, 10^9^/L	200.0 (167.0–242.0)	187.0 (154.0–221.0)	<0.001
White blood cell count, 10^9^/L	7.63 (6.37–9.50)	6.80 (5.73–8.10)	<0.001
Neutrophil, 10^9^/L	5.35 (4.27–7.00)	4.20 (3.47–5.28)	<0.001
Lymphocyte, 10^9^/L	1.41 (1.07–1.89)	1.69 (1.33–2.07)	<0.001
Monocyte, 10^9^/L	0.50 (0.40–0.61)	0.51 (0.40–0.68)	0.016
SII	808.43 (490.99–1,221.09)	466.64 (327.56–655.71)	<0.001
SIRI	2.15 (1.32–2.97)	1.30 (0.87–2.08)	<0.001
PIV	443.21 (263.55–634.68)	236.17 (149.58–407.62)	<0.001
Fasting glucose, mmol/L	7.02 (5.65–8.63)	6.60 (5.35–7.79)	0.002
HbA1c,%	7.10 (6.30–8.20)	7.00 (6.20–8.00)	0.001
Triglyceride, mmol/L	1.53 (1.08–2.11)	1.44 (1.07–1.99)	0.160
Total cholesterol, mmol/L	3.96 (3.22–4.81)	3.81 (3.14–4.62)	0.011
LDL-C, mmol/L	2.13 (1.46–2.95)	1.98 (1.35–2.75)	0.012
HDL-C, mmol/L	0.98 (0.85–1.13)	0.96 (0.83–1.12)	0.086
BUN, mmol/L	6.10 (4.90–7.80)	5.80 (4.80–7.00)	0.005
Creatinine, μmol/L	82.0 (69.0–98.0)	80.0 (68.0–93.0)	0.055
Uric acid, μmol/L	329.0 (273.0–395.0)	331.0 (278.0–398.0)	0.762
hsCRP, mg/L	2.90 (1.00–8.20)	2.56 (0.91–6.91)	0.058
eGFR, ml/min/1.73 m^2^	81.91 (65.21–95.13)	86.48 (71.83–96.93)	<0.001
Severity of CAD			
One-vessel disease	87 (14.1)	94 (11.9)	0.230
Two-vessel disease	168 (27.2)	210 (26.5)	0.809
Three-vessel disease	363 (58.7)	487 (61.6)	0.297
Medication			
ACE inhibitor/ARBs	349 (56.5)	438 (55.4)	0.705
β-blockers	443 (71.7)	554 (70.0)	0.517
Calcium channel blockers	190 (30.7)	216 (27.3)	0.173
Nitrates	243 (39.3)	336 (42.5)	0.230
Diuretic	127(20.6)	146(18.5)	0.342
Statins	556(90.0)	704(89.0)	0.601

CC, coronary collateralization; SBP, Systolic blood pressure; DBP, diastolic blood pressure; MI, myocardial infarction; SII, systemic immune-inflammation index; SIRI, systemic inflammation response index; PIV, pan-immune-inﬂammation value; HbA1c, glycosylated hemoglobin A1c; LDL-C, low-density lipoprotein cholesterol; HDL-C, high-density lipoprotein cholesterol; Lp(a), lipoprotein a; BUN, blood urea nitrogen; hsCRP, high-sensitivity C-reactive protein; eGFR, estimated glomerular filtration rate.

### SII, SIRI and PIV with coronary collateralization

**Figure 2:**
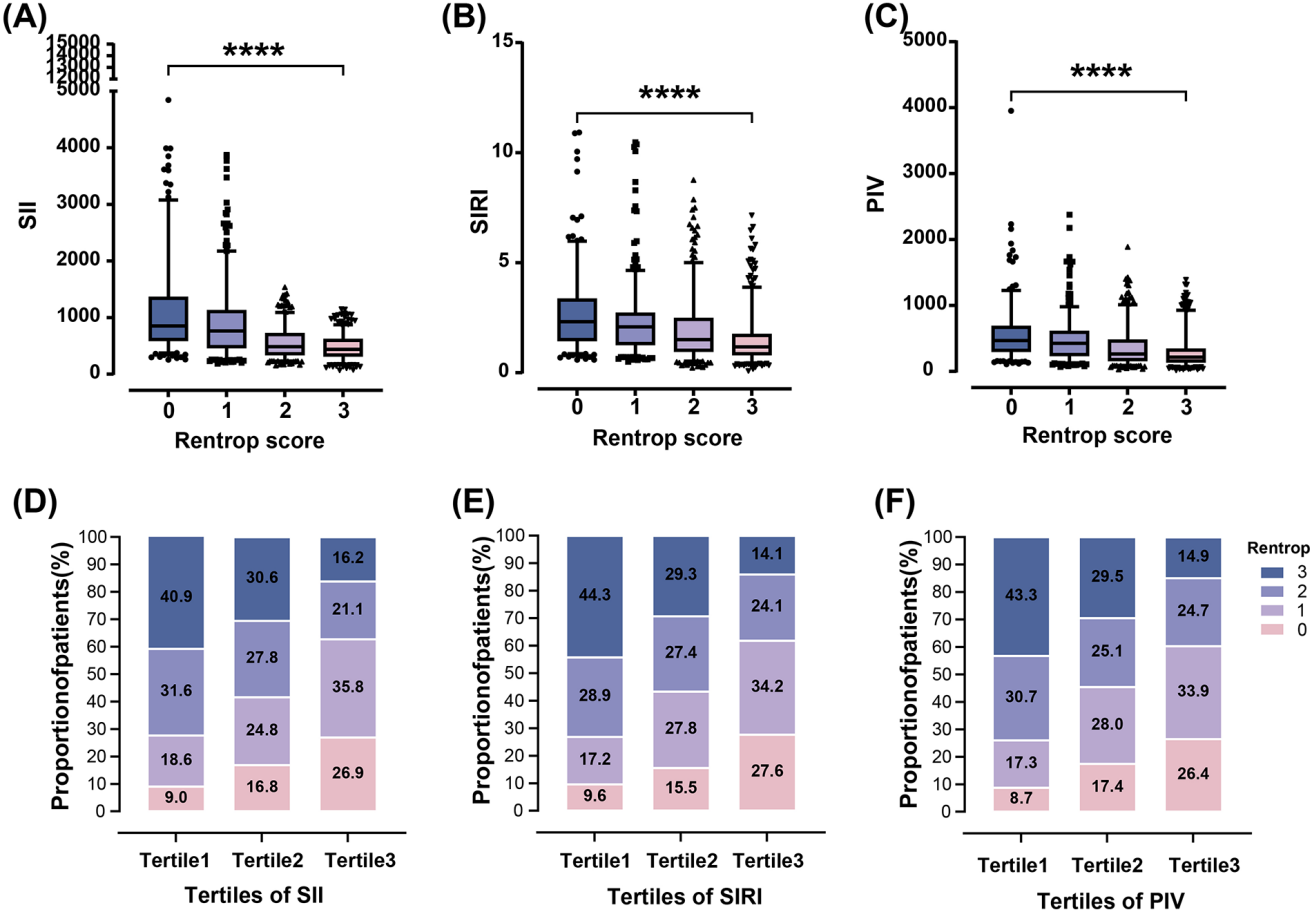
Relationship of SII, SIRI and PIV with rentrop score in T2DM patients with CTO. SII, SIRI and PIV decreased gradually across Rentrop score 0–3 [**(A)–(C)**, ****P < 0.0001]. The proportion of poor CC increased from the lowest tertile to the highest tertile of SII, SIRI and PIV **(D)–(F)**.

SII, SIRI and PIV levels increased stepwise across Rentrop score 0–3 (Figure 2), with significantly higher levels in patients with poor CC compared to those with good CC [SII: 808.43(490.99–1,221.09) vs. 466.64 (327.56–655.71); SIRI: 2.15(1.32–2.97) vs. 1.30 (0.87–2.08); PIV: 443.21(263.55–634.68) vs. 236.17(149.58–407.62), all *P* < 0.001] (Table 1). Increased SII tertiles (OR, 3.005; 95% CI, 2.589–3.489; *P* < 0.001), SIRI tertiles (OR, 2.111; 95% CI, 1.839–2.424; *P* < 0.001) and PIV tertiles (OR, 2.244; 95% CI, 1.951–2.581; *P* < 0.001) were associated with a higher proportion of poor CC, respectively (Figure 2).

Multivariate logistic regression analysis revealed that after adjustment for confounding factors including age, gender, BMI, hypercholesterolemia, hematological data, HbA1c and eGFR, high tertile of SII, SIRI and PIV levels remained independent predictors for poor CC in T2DM patients with CTO (Table 2).

**Table 2:** Multivariate analysis of risk factors for poor CC in T2DM patients with CTO.

	Variable	OR (95% CI)	*P* value
Model 1	Age (per 10 years)	1.319 (1.165–1.493)	<0.001
	Female	1.333 (0.992–1.790)	0.057
	Body mass index (per SD)	1.184 (1.049–1.336)	0.006
	Hypertension	1.205 (0.919–1.579)	0.177
	Hypercholesterolemia	1.555 (1.175–2.057)	0.002
	HbA1c (per SD)	1.179 (1.046–1.328)	0.007
	eGFR (per SD)	0.954 (0.841–1.083)	0.469
	SII (per tertile)	2.959 (2.537–3.451)	<0.001
Model 2	Age (per 10 years)	1.261 (1.121–1.419)	<0.001
	Female	1.630 (1.224–2.170)	0.001
	Body mass index (per SD)	1.152 (1.025–1.295)	0.017
	Hypertension	1.211 (0.934–1.571)	0.149
	Hypercholesterolemia	1.663 (1.271–2.177)	<0.001
	HbA1c (per SD)	1.238 (1.104–1.389)	<0.001
	eGFR (per SD)	0.945 (0.836–1.069)	0.372
	SIRI (per tertile)	2.146 (1.857–2.480)	<0.001
Model 3	Age (per 10 years)	1.304 (1.158–1.469)	<0.001
	Female	1.466 (1.103–1.951)	0.009
	Body mass index (per SD)	1.149 (1.021–1.293)	0.021
	Hypertension	1.198 (0.922–1.555)	0.176
	Hypercholesterolemia	1.588 (1.212–2.081)	0.001
	HbA1c (per SD)	1.197 (1.066–1.343)	0.002
	eGFR (per SD)	0.928 (0.820–1.049)	0.231
	PIV (per tertile)	2.230 (1.929–2.578)	<0.001

Abbreviations are as in Table 1.

**Figure 3:**
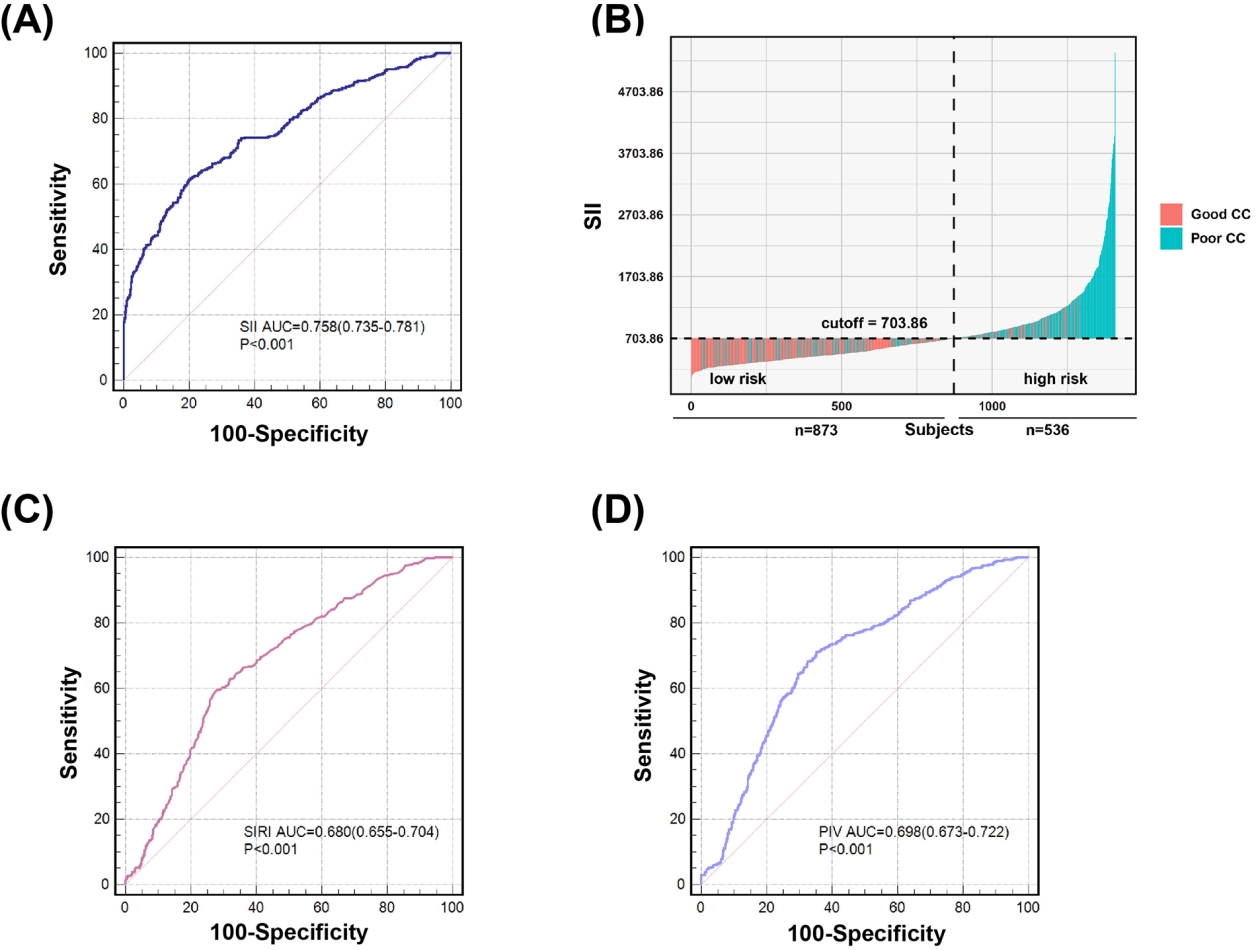
Value of SII **(A)** and **(B)**, SIRI **(C)** and PIV **(D)** for predicting poor coronary collateralization. According to the ROC analysis, SII showed better predictive ability of poor CC compared to SIRI and PIV. The likelihood that a cutoff value of SII 703.86 could accurately differentiate patients with poor CC from those with good CC was 75.8%, with 61.2% sensitivity and 80.1% specificity **(A)** and **(B)**. The likelihood that a cutoff value of SIRI 1.93 could accurately differentiate patients with poor CC from those with good CC was 68.0%, with 59.5% sensitivity and 72.1% specificity **(C)**. The likelihood that a cutoff value of PIV 299.64 could accurately differentiate patients with poor CC from those with good CC was 69.8%, with 71.2% sensitivity and 64.6% specificity **(D)**.

In ROC analysis, the likelihood that a cutoff value of SII 703.86 could accurately differentiate patients with poor CC from those with good CC was 75.8% (95% CI, 0.735–0.781), with 61.2% sensitivity and 80.1% specificity. SII had a significantly better predictive ability for poor CC than SIRI (AUC: 0.680, 95% CI, 0.655–0.704) and PIV (AUC: 0.698, 95% CI, 0.673–0.722) (all *P* < 0.001) (Figure 3).

### Influence of glycemic control

**Figure 4:**
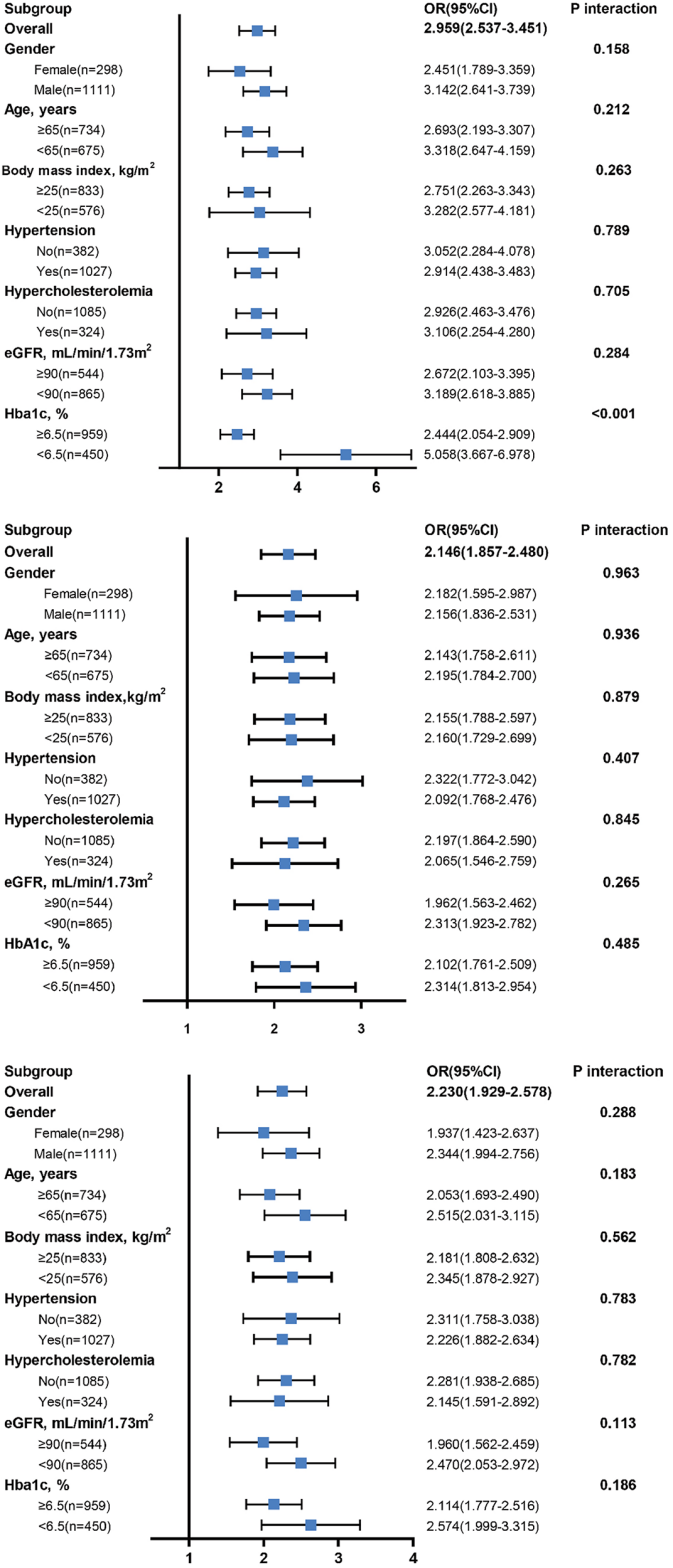
Diagnostic value of SII (top), SIRI (middle), and PIV (bottom) in subgroup analysis.

**Figure 5:**
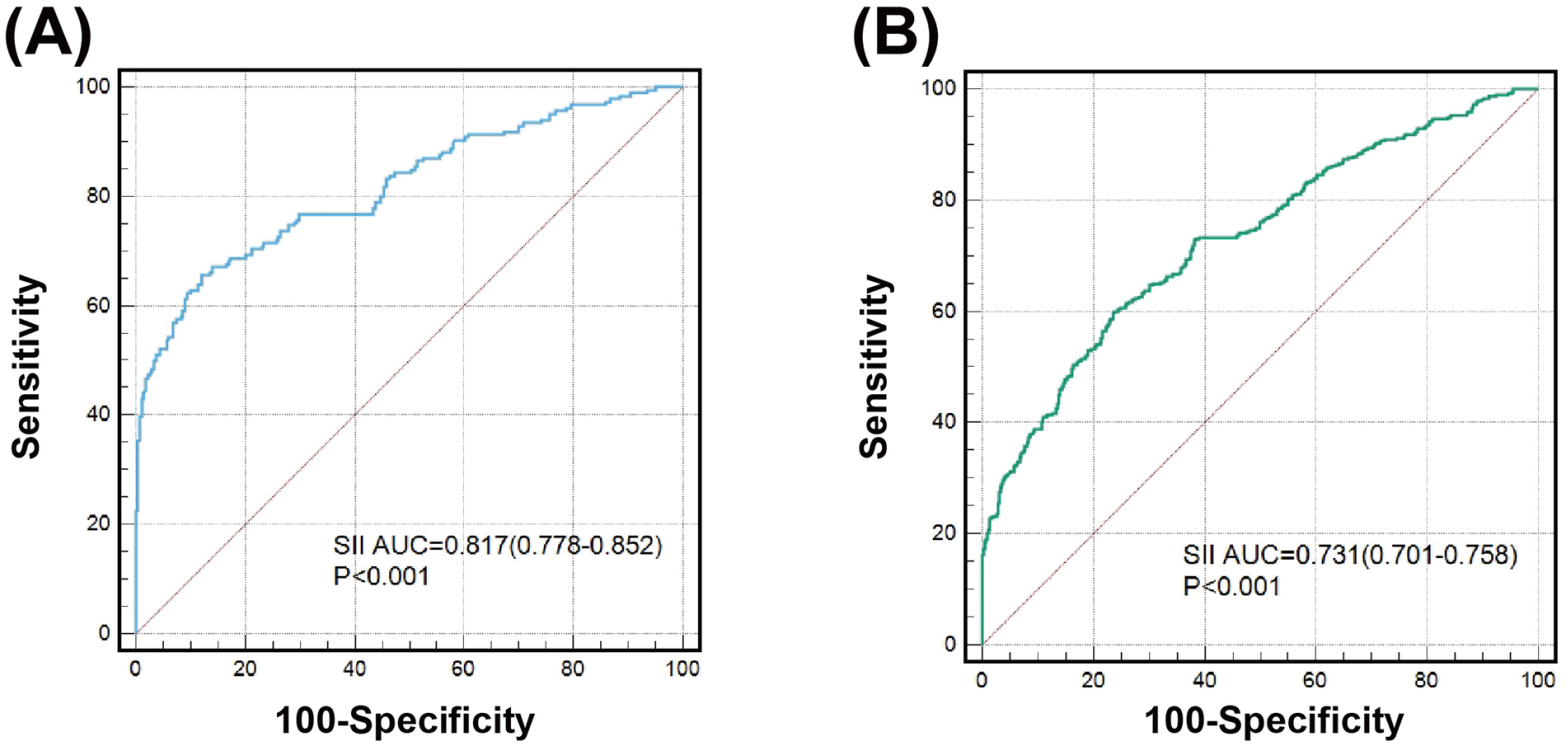
Value of SII for predicting poor CC in patients with good **(A)** or poor glycemic control **(B)**.

After stratifying the study population by age, gender, BMI, hypercholesterolemia, eGFR and HbA1c, increased SII, SIRI and PIV levels showed consistent predictive value for poor CC with odds ratio (OR) ranging from 1.937 to 5.058. No interactions of SII, SIRI and PIV with age, gender, BMI, hypertension, hypercholesterolemia, and eGFR on poor CC were observed (*P* for interaction >0.05). However, there existed a significant interaction between blood concentration of HbA1c and SII on poor CC (*P* for interaction <0.001). Compared to patients with HbA1c ≥ 6.5%, high SII values were associated with a greater risk (OR: 5.058 vs. 2.444) (Figure 4) and provided a better predictive ability (AUC: 0.817 vs. 0.731) for poor CC in those with HbA1c < 6.5% (Figure 5).

## Discussion

Our results showed that (1) systemic immune-inflammatory markers including SII, SIRI, and PIV were associated with angiographic CC in T2DM patients with CTO; (2) SII provided a significantly better predictive ability for poor CC than SIRI and PIV; (3) high SII levels conferred a greater risk and had a better prediction for poor CC in patients with HbA1c < 6.5% as compared to those with HbA1c ≥ 6.5%.

It is well recognized that collateral formation is impaired in T2DM patients and CTO, however, the underlying mechanism remains not fully understood. Previous studies have shown that chronic low-grade inflammation plays a central role in the pathophysiology of coronary collateral development (21). Patients with T2DM have a persistent inflammatory status, which could decrease production of nitric oxide and increase production of reactive oxygen species, leading to elevated oxidative stress and endothelial dysfunction (22–24). This supports the view that inflammation-related markers might provide predictive value on CC in T2DM patients with CTO. Several readily available inflammatory biomarkers including high-sensitivity C-reactive protein (hsCRP), neutrophil, monocyte, platelet (PLT), neutrophil to lymphocyte ratio (NLR), platelet to lymphocyte ratio (PLR) have been found to be associated with CC in patients with CTO (25–28). However, the inflammatory markers that are composed of a single component (neutrophil, lymphocyte, or platelet) and two components (NLR and PLR) are often affected by other confounding factors and become relatively weak predictors of prognosis. SII, SIRI and PIV are integrated markers of systemic immune-inflammation and their role in the evaluation of cardiovascular diseases has been recently studied (29–31). SII obtained by multiplying NLR and platelet increases in the presence of chronic inflammation, and it is a relatively strong inflammatory marker that may inhibit endothelial progenitor cell differentiation, survival, and function—key components of angiogenesis. SII was thought to be more reliable and representative of inﬂammation and thrombosis than PLR and NLR (32, 33). Dziedzic et al. demonstrated a relationship of SII values with the severity of coronary artery disease (34). Xia et al. reported that high SII was significantly associated with increased all-cause and cardiovascular mortality risks (9). In the present study, we found that SII levels correlated closely with Rentrop score in T2DM patients with CTO. The predictive ability of SII on poor CC performed well even after adjusting for confounding factors and was much better than other inflammation indexes (SIRI and PIV). Mechanistically, elevated SII levels indicated the increased neutrophil and platelet counts and decreased lymphocyte counts. Neutrophils release inflammatory cytokines and reactive oxygen species, which may exacerbate endothelial dysfunction and inhibit angiogenesis (35). Low levels of lymphocytes reflect immune suppression and are associated with impaired endothelial function (36). Platelets play a vital role in new blood vessel formation by involving many angiogenesis promoters and inhibitors (37). Thus, elevated SII levels are associated with systemic inflammation, immune dysregulation, and impaired angiogenic processes. However, SIRI focuses more on monocytes and does not account for platelets, which is essential in angiogenesis and vascular remodeling. Although PIV includes platelet counts, the weights of the component are linearly distributed, which may obscure the dominant role of platelets. Taken together, it may explain why SII predicts poor CC more effectively than SIRI and PIV in T2DM patients with CTO.

In addition, the diagnostic performance of SII remained satisfied in different subgroup analysis. Interestingly, using HbA1c 6.5% as the threshold of glycemic control, a significant interaction was identified when we analyzed the predictive value of SII on poor CC. High SII was associated with a greater risk and provided a significantly better prediction for poor CC in patients with HbA1c < 6.5%, as compared to those with HbA1c > 6.5%. The reason for that remains unclear, but our findings are, at least partly, supportive of previous reports which emphasize the relationship between inflammation and the development of coronary collaterals (10, 12). We speculate that in T2DM patients with CTO and normal glycemic control, the systemic inflammatory condition is in relatively low grade. An increase in SII value could be more reliable indicator of a great risk for developing poor CC. In contrast, patients with poor glycemic control often have a high grade of inflammatory status as suggested by high baseline SII levels even in those with good CC, thus the effect of changes of SII were less remarkable for forecasting the risk of poor CC.

### Study limitations

The present study had several limitations. First, this was a single center retrospective and observational study, thus the selected population may not represent the whole aimed cohort, and the causal link of SII, SIRI and PIV with poor CC was not detected. Second, although several traditional risk factors were considered, there were still some confounding factors that were not included in the analysis. Third, the degree of coronary collateral circulation was estimated according to the Rentrop scoring system, whereas measurement of collateral flow index may be more accurate.

## Conclusion and perspective

This study demonstrates that SII, SIRI, and PIV are closely associated with angiographic CC, and SII provides a better prediction for poor CC than SIRI and PIV in T2DM patients with CTO, especially at good glycemic control. The good predictive ability of SII on poor CC validated its potential as a diagnostic biomarker in T2DM patients with CTO. Since there is increasing evidence that treatment decisions and indications for recanalization of a CTO should be based not only on clinical characteristics and occluded lesion morphology, but also on collateral quality and myocardial viability (38–41). SII shows CC status as a useful, simple, easily measurable, and cheap indicator, and could be used as a reference for clinicians to improve the care of diabetic patients with stable coronary artery disease. Further prospective studies with large sample size and long-term clinical follow-up are warranted to prove the diagnostic and prognostic value of SII, in relation to CC, for patients with T2DM and CTO.

## Data Availability

The original contributions presented in the study are included in the article/Supplementary Material, further inquiries can be directed to the corresponding author.
